# Supplementary material for: Maternal characteristics associated with referral to obstetrician-led care in low-risk pregnant women in the Netherlands: A retrospective cohort study
Source: PLoS One. 2023 Mar 15;18(3):e0282883. doi: 10.1371/journal.pone.0282883 (PMC10016726; doi:10.1371/journal.pone.0282883)
Supplement: S1 Table — (DOCX) [file pone.0282883.s001.docx]

**Table S1: Association between missing data in characteristics and referral.**

|  | Nulliparous  OR (95%CI) | Multiparous  OR (95% CI) |
| --- | --- | --- |
| Antepartum | 0.86 (0.46-1.57) | 1.34 (0.81-2.21) |
| Intrapartum | 1.81 (0.81-4.50) | 2.52 (1.25-5.13) |
