# Supplementary material for: Maternal characteristics associated with referral to obstetrician-led care in low-risk pregnant women in the Netherlands: A retrospective cohort study
Source: PLoS One. 2023 Mar 15;18(3):e0282883. doi: 10.1371/journal.pone.0282883 (PMC10016726; doi:10.1371/journal.pone.0282883)
Supplement: S2 Table — (DOCX) [file pone.0282883.s002.docx]

| **Table S2 a & b: Association with referral split by parity and period (original data).** | | |  | |  | |
| --- | --- | --- | --- | --- | --- | --- |
|  |  |  | |  | |  |
| *Table S2a: Association with referral antepartum (n = 1096)* | |  | |  | |  |
| **Variable** | **Nulliparous** | | | **Multiparous** | | |
|  | *Full model* | *Final model* | | *Full model* | | *Final model* |
|  | OR (95%CI) | OR (95%CI) | | OR (95%CI) | | OR (95%CI) |
|  |  |  | |  | |  |
| Age (years) | 1.08 (1.03 - 1.13) | **1.09 (1.04 - 1.13)** | | 1.00 (0.96 - 1.04) | |  |
|  |  |  | |  | |  |
| BMI (kg/m^2^) |  |  | |  | |  |
| <18.5 | 0.46 (0.19 - 1.12) | **0.48 (0.20 - 1.15)** | | 0.36 (0.13 - 1.03) | | **0.36 (0.13 - 1.00)** |
| 18.5-24.9 | 1.00 | 1.00 | | 1.00 | | 1.00 |
| 25.0-29.9 | 2.04 (1.28 - 3.26) | **2.05 (1.30 - 3.25)** | | 0.91 (0.59 - 1.40) | | **0.92 (0.60 - 1.40)** |
| ≥ 30.0 | 2.71 (1.40 - 5.22) | **2.64 (1.39 - 5.03)** | | 1.58 (0.91 - 2.75) | | **1.62 (0.93 - 2.80)** |
|  |  |  | |  | |  |
| Preconception period (1 year ≤ / > 1 year) | 1.22 (0.68 - 2.19) |  | | 1.77 (0.82 - 3.82) | |  |
|  |  |  | |  | |  |
| Education level |  |  | |  | |  |
| low | 1.00 |  | | 1.00 | | 1.00 |
| medium | 0.85 (0.41 - 1.74) |  | | 0.78 (0.44 - 1.38) | | **0.78 (0.45 - 1.34)** |
| high | 1.03 (0.48 - 2.19) |  | | 0.53 (0.29 - 0.98) | | **0.49 (0.27 - 0.89)** |
|  |  |  | |  | |  |
| Employment (no/yes) | 1.12 (0.68 - 1.87) |  | | 1.59 (1.04 - 2.45) | |  |
|  |  |  | |  | |  |
| Ethnicity (Dutch / Non-Dutch) | 0.98 (0.63 - 1.52) |  | | 1.08 (0.72 - 1.63) | |  |
|  |  |  | |  | |  |
| Deprivation (no/yes) | 1.92 (1.14 - 3.25) | **1.84 (1.12 - 3.00)** | | 1.40 (0.87 - 2.25) | |  |
|  |  |  | |  | |  |
| Smoking (no/yes) | 1.05 (0.65 - 1.68) |  | | 1.07 (0.67 - 1.70) | |  |
|  |  |  | |  | |  |
| Psychological problems (no/yes) | 0.87 (0.53 - 1.42) |  | | 1.23 (0.80 - 1.90) | |  |
|  |  |  | |  | |  |
| Sexual violence (no/yes) | 1.60 (0.84 - 3.06) |  | | 1.35 (0.73 - 2.48) | |  |
|  |  |  | |  | |  |
| Consultation obstetric care |  |  | |  | |  |
| none | 1.00 |  | | 1.00 | | 1.00 |
| 1 | 0.97 (0.61 - 1.54) |  | | 0.62 (0.40 - 0.97) | | **0.65 (0.42 - 1.01)** |
| > 1 | 0.97 (0.58 - 1.61) |  | | 0.62 (0.39 - 0.98) | | **0.62 (0.39 - 0.97)** |
|  |  |  | |  | |  |
|  |  |  | |  | |  |
|  |  |  | |  | |  |
|  |  |  | |  | |  |
|  |  |  | |  | |  |
|  |  |  | |  | |  |
|  |  |  | |  | |  |
| *Table S2b: Association with referral intrapartum (n = 617)* | |  | |  | |  |
| Variable | **Nulliparous** | | | **Multiparous** | | |
|  | *Full model* | *Final model* | | *Full model* | | *Final model* |
|  | OR (95%CI) | OR (95%CI) | | OR (95%CI) | | OR (95%CI) |
|  |  |  | |  | |  |
| Age (years) | 1.02 (0.95 - 1.09) |  | | 1.02 (0.96 - 1.08) | |  |
|  |  |  | |  | |  |
| BMI (kg/m^2^) |  |  | |  | |  |
| <18.5 | 1.77 (0.68 - 4.59) |  | | 0.82 (0.27 - 2.50) | |  |
| 18.5-24.9 | 1.00 |  | | 1.00 | |  |
| 25.0-29.9 | 1.02 (0.52 - 2.03) |  | | 0.63 (0.34 - 1.19) | |  |
| ≥ 30.0 | 1.17 (0.39 - 3.51) |  | | 0.89 (0.38 - 2.11) | |  |
|  |  |  | |  | |  |
| Preconception period (1 year ≤ / > 1 year) | 0.36 (0.16 - 0.83) | **0.40 (0.18 - 0.88)** | | 0.23 (0.03 - 1.93) | |  |
|  |  |  | |  | |  |
| Education level |  |  | |  | |  |
| low | 1.00 |  | | 1.00 | |  |
| medium | 2.39 (0.97 - 5.88) |  | | 1.46 (0.61 - 3.52) | |  |
| high | 2.35 (0.89 - 6.24) |  | | 0.82 (0.32 - 2.12) | |  |
|  |  |  | |  | |  |
| Employment (no/yes) | 0.81 (0.42 - 1.58) |  | | 1.28 (0.70 - 2.36) | |  |
|  |  |  | |  | |  |
| Ethnicity (Dutch / Non-Dutch) | 1.03 (0.59 - 1.82) |  | | 1.81 (1.01 - 3.26) | | **1.61 (0.95 - 2.72)** |
|  |  |  | |  | |  |
| Deprivation (no/yes) | 0.88 (0.42 - 1.83) |  | | 1.18 (0.59 - 2.35) | |  |
|  |  |  | |  | |  |
| Smoking (no/yes) | 1.16 (0.61 - 2.18) |  | | 0.68 (0.34 - 1.37) | |  |
|  |  |  | |  | |  |
| Psychological problems (no/yes) | 1.05 (0.55 - 1.97) |  | | 1.01 (0.54 - 1.86) | |  |
|  |  |  | |  | |  |
| Sexual violence (no/yes) | 0.39 (0.16 - 0.98) | **0.42 (0.19 - 0.96)** | | 1.67 (0.69 - 4.05) | |  |
|  |  |  | |  | |  |
| Consultation obstetric care |  |  | |  | |  |
| none | 1.00 |  | | 1.00 | | 1.00 |
| 1 | 0.93 (0.50 - 1.72) |  | | 1.47 (0.77 - 2.78) | | **1.45 (0.78 - 2.68)** |
| > 1 | 1.29 (0.65 - 2.55) |  | | 1.90 (0.99 - 3.65) | | **2.06 (1.11 - 3.82)** |
