# Supplementary material for: Maternal characteristics associated with referral to obstetrician-led care in low-risk pregnant women in the Netherlands: A retrospective cohort study
Source: PLoS One. 2023 Mar 15;18(3):e0282883. doi: 10.1371/journal.pone.0282883 (PMC10016726; doi:10.1371/journal.pone.0282883)
Supplement: S1 File — (DOCX) [file pone.0282883.s003.docx]

STROBE Statement—checklist of items that should be included in reports of observational studies

**Maternal characteristics associated with referral to obstetrician-led care in low-risk pregnant women in the Netherlands: a retrospective cohort study**

*Susan Niessink – Beckers, Corine J. Verhoeven, Marleen J. Nahuis, Lisanne A. Horvat-Gitsels, Janneke T. Gitsels – van der Wal*

|  | | | Item No. | Recommendation | Page  No. | | | | | Relevant text from manuscript |
| --- | --- | --- | --- | --- | --- | --- | --- | --- | --- | --- |
| **Title and abstract** | | | 1 | (*a*) Indicate the study’s design with a commonly used term in the title or the abstract | 1 | | | | | a retrospective cohort study |
|  |  |  |  | (*b*) Provide in the abstract an informative and balanced summary of what was done and what was found | 2-3 | | | | | See abstract |
| Introduction | | | | | | | | | |  |
| Background/rationale | | | 2 | Explain the scientific background and rationale for the investigation being reported | 3-4 | | | | | “Multiple … outcomes” |
| Objectives | | | 3 | State specific objectives, including any prespecified hypotheses | 4 | | | | | “Awareness … care” |
| Methods | | | | | | | | | |  |
| Study design | | | 4 | Present key elements of study design early in the paper | 4-5 | | | | | “The retrospective …. their presence ” |
| Setting | | | 5 | Describe the setting, locations, and relevant dates, including periods of recruitment, exposure, follow-up, and data collection | 4 | | | | | “The retrospective …. January 2017” |
| Participants | | | 6 | (*a*) *Cohort study*—Give the eligibility criteria, and the sources and methods of selection of participants. Describe methods of follow-up  *Case-control study*—Give the eligibility criteria, and the sources and methods of case ascertainment and control selection. Give the rationale for the choice of cases and controls  *Cross-sectional study*—Give the eligibility criteria, and the sources and methods of selection of participants | 4-5 | | | | | “The study … their presence” |
|  |  |  |  | (*b*) *Cohort study*—For matched studies, give matching criteria and number of exposed and unexposed  *Case-control study*—For matched studies, give matching criteria and the number of controls per case |  | | | | | inapplicable |
| Variables | | | 7 | Clearly define all outcomes, exposures, predictors, potential confounders, and effect modifiers. Give diagnostic criteria, if applicable | 5-6 | | | | | Subhead: Measures  “The outcomes …of birth” |
| Data sources/ measurement | | | 8* | For each variable of interest, give sources of data and details of methods of assessment (measurement). Describe comparability of assessment methods if there is more than one group | 5-6 | | | | | Subhead: Measures  “The outcomes …of birth” |
| Bias | | | 9 | Describe any efforts to address potential sources of bias | 6 | | | | | “Missing data …multiple imputations” |
| Study size | | | 10 | Explain how the study size was arrived at | 4-5 | | | | | “The study … postnatal care” |
| Quantitative variables | | 11 | | Explain how quantitative variables were handled in the analyses. If applicable, describe which groupings were chosen and why | | 5 | | | *“*Dichotomous variables … date of birth” | |
| Statistical methods | | 12 | | (*a*) Describe all statistical methods, including those used to control for confounding | 6 | | | “Missing data …by parity” | | |
|  |  |  |  | (*b*) Describe any methods used to examine subgroups and interactions | 6 | | | The study population's baseline characteristics were summarised by means and standard deviations for normally distributed continuous variables, and frequencies and percentages for categorical variables, including dichotomous ones | | |
|  |  |  |  | (*c*) Explain how missing data were addressed | 6 | | | Missing data patterns were explored by fitting logistic regression models to understand potential selection bias and dealt with by multiple imputations | | |
|  |  |  |  | (*d*) *Cohort study*—If applicable, explain how loss to follow-up was addressed  *Case-control study*—If applicable, explain how matching of cases and controls was addressed  *Cross-sectional study*—If applicable, describe analytical methods taking account of sampling strategy | 6 | | | Missing data patterns were explored by fitting logistic regression models to understand potential selection bias and dealt with by multiple imputations | | |
|  |  |  |  | (*e*) Describe any sensitivity analyses | 7 | | | The sensitivity analysis included repeating the model fittings on the subset with complete information (i.e. complete case analysis) | | |
| Results | | | | | | | | | | |
| Participants | | 13* | | (a) Report numbers of individuals at each stage of study—eg numbers potentially eligible, examined for eligibility, confirmed eligible, included in the study, completing follow-up, and analysed | 6 | | | The study …multiparous women. | | |
|  |  |  |  | (b) Give reasons for non-participation at each stage | 6 | | | The study …multiparous women. | | |
|  |  |  |  | (c) Consider use of a flow diagram |  | | | Comment reviewer: remove the flow diagram | | |
| Descriptive data | | 14* | | (a) Give characteristics of study participants (eg demographic, clinical, social) and information on exposures and potential confounders | 6 | | | “The study … Table 1” | | |
|  |  |  |  | (b) Indicate number of participants with missing data for each variable of interest | 9 | | | There was limited missing data (<1%), except for education (3%) and preconception period (7%), with overall 11% missing observations. | | |
|  |  |  |  | (c) *Cohort study*—Summarise follow-up time (eg, average and total amount) |  | | | inapplicable | | |
| Outcome data | | 15* | | *Cohort study*—Report numbers of outcome events or summary measures over time |  | | | Table 1 & 2 | | |
|  |  |  |  | *Case-control study—*Report numbers in each exposure category, or summary measures of exposure |  | | |  | | |
|  |  |  |  | *Cross-sectional study—*Report numbers of outcome events or summary measures |  | | |  | | |
| Main results | | 16 | | (*a*) Give unadjusted estimates and, if applicable, confounder-adjusted estimates and their precision (eg, 95% confidence interval). Make clear which confounders were adjusted for and why they were included | 10-13 | | | “The full … were smokers (0.75; 0.57-0.97)”. | | |
|  |  |  |  | (*b*) Report category boundaries when continuous variables were categorized | 5 | | | BMI and number of consultations with an obstetrician were categorised due to non-normal distributions. Underweight was defined as having a BMI of <18.5, healthy weight was defined as a BMI of 18.5-24.9 , overweight was defined as a BMI of 25.0-29.9, and obesity was defined as a BMI of ≥30.0 [30]. The number of consultations with an obstetrician --a standalone consult in obstetric care without women discontinuing midwife-led care-- was defined as none, one, and ≥2. | | |
|  |  |  |  | (*c*) If relevant, consider translating estimates of relative risk into absolute risk for a meaningful time period |  | | | inapplicable | | |
| Other analyses | 17 | | Report other analyses done—eg analyses of subgroups and interactions, and sensitivity analyses | | 13 | | Their model performances in Table S2 in Additional File 2 | | | |
| Discussion | | | | | | | | | | |
| Key results | 18 | | Summarise key results with reference to study objectives | | 13 | | “our study … obstetrician-led care” | | | |
| Limitations | 19 | | Discuss limitations of the study, taking into account sources of potential bias or imprecision. Discuss both direction and magnitude of any potential bias | | 15 | | Subhead: Strength and limitations | | | |
| Interpretation | 20 | | Give a cautious overall interpretation of results considering objectives, limitations, multiplicity of analyses, results from similar studies, and other relevant evidence | | 13-15 | | “In agreement … the associations” | | | |
| Generalisability | 21 | | Discuss the generalisability (external validity) of the study results | | 15 | | Therefore, we cannot generalise our results to all low-risk women in the Netherlands. | | | |
| Other information | | |  | | | | | | | |
| Funding | 22 | | Give the source of funding and the role of the funders for the present study and, if applicable, for the original study on which the present article is based | |  | | No funding | | | |

*Give information separately for cases and controls in case-control studies and, if applicable, for exposed and unexposed groups in cohort and cross-sectional studies.

**Note:** An Explanation and Elaboration article discusses each checklist item and gives methodological background and published examples of transparent reporting. The STROBE checklist is best used in conjunction with this article (freely available on the Web sites of PLoS Medicine at http://www.plosmedicine.org/, Annals of Internal Medicine at http://www.annals.org/, and Epidemiology at http://www.epidem.com/). Information on the STROBE Initiative is available at www.strobe-statement.org.
