# Supplementary material for: Maternal characteristics associated with referral to obstetrician-led care in low-risk pregnant women in the Netherlands: A retrospective cohort study
Source: PLoS One. 2023 Mar 15;18(3):e0282883. doi: 10.1371/journal.pone.0282883 (PMC10016726; doi:10.1371/journal.pone.0282883)
Supplement: Attachment — Submitted filename: Review PONE-D-21-28982_april 2022.docx [file pone.0282883.s004.docx]

An interesting paper that could feed the discussion on the quality of risk selection during pregnancy and birth in the Netherlands. Albeit with caution as the results are based on the data of one midwifery practice alone.

My main problem lies in the formulation of the research question. As I am not sure it is framed correctly.

The paper would benefit from textual and English language editing

Please find more detailed remarks and suggestions below:

Abstract

P2

L25 Rather bold opening statement, which might not be strictly true if this is meant world-wide. Perhaps add region / country.

L27 Risk assessment is not only applied at the beginning of pregnancy but also during each prenatal check-up as well as during labour and post-partum period.

L27 Slightly vague: you mean maternal characteristics that influence referrals?

L30 ‘indicate’ should be ‘influence’ or do you really mean a characteristic could be an indication for referral?

L32 ‘a midwifery practice’ > better ‘one midwifery practice’

L33 I am not sure what an outcome referral is, perhaps you mean primary outcome or outcome of referral(s)?

L35 indicators for?

L39 obesity instead of obese

L41 sexual abuse instead of sexual violence

L44 consultations with? The midwife? Obstetrician? (L48 / L50same)

L52-56 Conclusions: I think this conclusion cannot be made on the basis of this study and these results. The results show that certain characteristics seem to be associated with an increased referral rate but do not show if the referral was justified based on these characteristics. Although it could be argued that a referral of some sort might be needed or better more care and support, it does not necessarily mean an obstetric referral is the answer in order to improve outcomes. Therefore to conclude that these characteristics are an indicator for referral to secondary care is not correct. The study does not show whether a referral to secondary care on the basis if these characteristics influence outcomes of pregnancy.

What’s more I think caution with the conclusion, also or may be even more so in the abstract, would be prudent as the results are based on the data of one midwifery practice only and as we know from earlier research, referral rates vary greatly in the Netherlands.

Introduction

L61: First sentence does not add anything: can be removed. Instead provide a bit more information on the different roles that midwives can have or the different systems that can be found.

L73 Please add some information on referrals for pain relief. It will not be clear to all readers that the need for pharmacological pain relief is a reason for referral in the Netherlands and that the need / wish for this has been rising and therefore influences referral numbers.

L77 C-section > please write out in full at first use

L86-88 I am having problems with the word ‘indicator’. I am not certain what the authors mean by this: do they mean to investigate whether certain characteristics were used as an indicator for / of referral? Or did they investigate if certain characteristics gave women a higher chance of referral? Or if there were certain characteristics associated with referral from midwifery- to obstetric lead care? Or did the authors want to investigate whether certain characteristics are *predictors* for referral?

After reading the main analysis section, I think what is meant is characteristics which were associated with a lower / higher chance of referral. In that case: please adjust the research question.

Please adjust the title of the manuscript accordingly.

Methods

L94 I believe the word ‘received’ should perhaps be replaced by ‘continued primary care after the first / booking visit at the midwives practice’ , unless the sample also includes women who were referred from secondary to primary care at a later stage?

L96/97 I am not sure what is meant by ‘noted in their files under their supervision’?

L112 ‘summarised by’, should be ‘categorized in’ and reasons referred were (also?) based on the LOI?

L114 Could you add some information on how these data were obtained? Ie by a questionnaire? Did the midwife ask this a standard question during the booking visit? How was ethnicity, deprivation defined? And preconception period, ie what constitutes a preconception period of longer than a year, how or by whom defined?

L120 ‘BMI was defined as underweight’ > should be phrased the other way around: Underweight was defined as a BMI < etc etc.

L123 maternal age based on DoB?

L142-146 please add that the data of 31 women were missing.

Table 1a & 1b: this shows the presence of certain characteristics in the group of women who entered primary care (1a) and women who started labour in primary care (1b) and you show the women with those characteristics who were not referred and who were referred: How ever you did not test for statistical differences between those groups. With other words: I would like to know if there was a difference in the chosen characteristics between the women who were referred and those who were not.

L154-156 please add the stats for the comparison

Tables 2a & 2b: Please add a description of the outcomes that are written in bold text ( I think it means the outcomes that are significant: in that case, please check for mistakes)

Discussion

L204-206 the description of what the study investigated here is not in line with the research question.

L240 perinatal outcomes: which ones: positive or negative ones?

L250-251 please move information on data collection to the methods section (also see earlier remark)

L285 Our study shows that maternal characteristics > there are words missing > *the effect of certain* maternal characteristics etc

Since the study was only performed in one midwifery practice and since we know that referral rates differ greatly between practices and regions, I would expect some reflection on this in the discussion. Possibly the characteristics of the midwives were of greater influence than those of the women…

Conclusion

288-291 I think this part of the conclusion should be worded more carefully as it is not clear that using these characteristics to determine / or screen for risk, and referring women to secondary care on the basis of this, improves outcomes.

Women with characteristics such as deprivation, unemployment, a history of sexual abuse might for example benefit from a different type of referral than that to secondary care.

Figures

I don’t think figure 1 adds anything to the paper: I would leave it out.

2a: since the item ‘other’ is in 4^th^ place: could you elaborate on this? The number of referrals for gestational diabetes seem quite large: how does this compare to (inter-) national numbers?

2b: I am surprised to see coagulation disorders and Uterus myomatosus as reasons for referral during labour as opposed to during pregnancy?
