# Supplementary material for: Maternal characteristics associated with referral to obstetrician-led care in low-risk pregnant women in the Netherlands: A retrospective cohort study
Source: PLoS One. 2023 Mar 15;18(3):e0282883. doi: 10.1371/journal.pone.0282883 (PMC10016726; doi:10.1371/journal.pone.0282883)
Supplement: Attachment — Submitted filename: Response to Reviewers.docx [file pone.0282883.s005.docx]

June 10, 2022

Manuscript ID: PONE-D-21-28982

Dear Editor and reviewers,

Thank you for the expert review of our manuscript titled ‘Maternal characteristics associated with referral to obstetrician-led care in low-risk pregnant women in the Netherlands: a retrospective cohort study’.

We want to thank you and the reviewers for the positive and constructive comments on our paper. We have used the reviewers' comments to improve our paper further, and we are pleased to send you our revised manuscript. The reviewers' comments and our response after each comment is presented in the table below. The actual changes in the paper are presented in track changes.

Sincerely, on behalf of all authors,

Susan Niessink-Beckers

| **Reviewers’ comment** | **Authors’ response** | **Changes in manuscript with tracked changes** |
| --- | --- | --- |
| *Editor*  PLOS ONE recommends the use of checklists such as the Strobe checklist for cohort studies and the RECORD checklist for REporting of studies Conducted using Observational Routinely-collected Data. | For our cohort study, we completed the Strobe checklist. It is available in the supporting information (S1 File). |  |
| *Reviewer 1* | | |
| First, I express my concern about the general purpose of this article. By using the word ‘indicator’, and by presenting sensitivity and specificity, the authors suggest that they want to predict referrals based on medical and non-medical characteristics, in order to support midwives in decisions on referrals (so called ‘risk assessment’). However, I doubt whether better prediction of referrals benefits the care for women. In the Dutch context, a referral to secondary care is needed if a problem arises that need attention of an obstetric professional. Even when a woman is at elevated risk for obstetric problems based on her psycho-social or lifestyle characteristics, an actual referral is needed only when this obstetric problem becomes evident. The prediction can be false, (total accuracy is lower than 70% in table 3), even if sensitivity and specificity are improved. Predicting a referral and acting on it by referring a woman based on the prediction creates a self-fulfilling prophecy. In my opinion, the strength of this article is not that it may contribute to better prediction or risk assessment, but that it shows once more that certain non-medical issues have impact on the course of pregnancy and birth. These issues need the attention of the midwife, mainly because these issues are in itself important for the well-being of the mother and her family. The midwife can support women with these issues, or she eventually can suggest the help of a psychologist, dietitian or welfare worker. In my opinion, this should be discussed more extensively in the section on implications for practice (Line 264), instead of the current focus on improving risk assessment. For instance, it would be interesting to know how the midwifery practice in this study deals with such issues or what they think is necessary to improve midwifery care for women with psycho-social or life-style issues. | We have changed the words 'indicator' and 'predictor' to 'association’ and have shifted the focus of the paper from improving risk assessment to raising awareness that non-medical factors can also affect referral and outcomes of pregnancy and childbirth and therefore deserve attention from health professionals.  We have made the following adjustments to improve our paper:  *“This exploratory study showed that several non-medical maternal characteristics of low-risk pregnant women are associated with referral from midwife-led to obstetrician-led care, and how these differ by parity and partum period.”*  *“Awareness of all maternal characteristics, including non-medical factors affecting the chance of a referral, will help healthcare professionals in providing individualised preventive care. To be able to intervene on those factors, we need to know which kind of non-medical factors are increasing a referral to obstetrician-led care. Therefore, our study investigated which non-medical maternal characteristics are associated with women’s referral from midwife-led to obstetrician-led care in the antepartum and intrapartum period.”*  *“Our study showed that multiple maternal characteristics are associated with a referral to obstetrician-led care. Especially, non-medical characteristics are associated with a referral whereas these remain underutilised in maternity care. It shows once more that certain non-medical issues can affect the course of pregnancy and birth. These matters need the attention of the midwife, mainly because they are in themselves important for the well-being of the mother and her family. Lack of social support is associated with adverse perinatal outcomes and should be considered as an opportunity to intervene on psychosocial and lifestyle maternal characteristics. Centering Pregnancy (group sessions) facilitates social support and enables women to improve self-confidence. The midwife can support women with these issues or can suggest the help of a psychologist, dietitian or welfare worker. Also, continuous care might be beneficial for these non-medical maternal characteristics, particularly case-load midwifery care is associated with a lower referral rate (56). We, therefore, advocate further research into the relation between non-medical maternal characteristics and referral, as well as how midwives can improve midwifery care for women with psychosocial or lifestyle issues (non-medical characteristics)..”*  *Added references:*  *Kweekel L, Gerrits T, Rijnders M, Brown P. The Role of Trust in CenteringPregnancy: Building Interpersonal Trust Relationships in Group-Based Prenatal Care in The Netherlands. Birth. 2017 Mar;44(1):41-47. doi: 10.1111/birt.12260. Epub 2016 Nov 7. PMID: 28198099.*  *Offerhaus, P., Jans, S., Hukkelhoven, C. et al. Women’s characteristics and care outcomes of caseload midwifery care in the Netherlands: a retrospective cohort study. BMC Pregnancy Childbirth* ***20,*** *517 (2020). https://doi.org/10.1186/s12884-020-03204-3* | Page 3, lines 59-61  Page 5, lines 101-103  Page 21, lines 318-334 |
| Selective group in intrapartum care You present the association of characteristics with antepartum referrals and with intrapartum referral. Every woman that experienced an antepartum referral, is no longer in midwifery care and therefor is no longer ‘at risk’ for an intrapartum referral. As the antepartum referrals are associated with certain characteristics, the intrapartum group (table 1b) has a different composition. For instance, in the antepartum group 12,5% of women have a BMI > 30, and in the intrapartum group this is 9.1%. This selection process may partly explain some of your results in the intrapartum analyses, as you state in Line 219 for the characteristic ‘preconception period > 1yr’. But also education levels (Line 231) and sexual violence (Line 236) show unexpected results. Perhaps because the women with the most serious impact on their health were already referred in the antepartum period? Please elaborate on this in the discussion. | Thank you for this remark. The characteristics of the group of antepartum and intrapartum women are indeed different, for the reason mentioned by the reviewer. We have elaborated on this in the discussion part.  *“**Overall, for the antepartum and intrapartum periods, the associations between maternal characteristics and a referral differ. Most of the characteristics in the antepartum and intrapartum periods are comparable, except for BMI≥ 30; in the antepartum period, 13% of the women have a BMI ≥ 30 compared to 9% of women in the intrapartum period. Since BMI ≥ 30 is associated with more complications during pregnancy these women might already have been referred to obstetrician-led care, which could explain the difference in the associations.”* | Page 19, lines 285-290 |
| You elaborate on the mixed / reverse relationship in the antepartum model for women with one or more consultations. Multiparous women with one or more consultations with obstetricians had a lower chance on an antepartum referral. That is contra-intuitive because consultations indicate that some kind of medical problem was present. Can this be explained by the way consultation are registered? Consultations that end up in a referral are probably not counted as a consultation but as a referral. If that is the case, please explain how this result should be interpreted in the antepartum analysis. In the intrapartum analyses the use of this variable make sense: it indicates that there was some kind of (medical) problem, but not severe enough to arrange a referral to obstetric care. | The registration of a consultation during the antepartum and intrapartum periods are similar. Consultations are defined as standalone consultations in obstetric care without women discontinuing midwife-led care. A consultation can be arranged for medical problems during this pregnancy, as well as for a problem in the previous pregnancy/pregnancies. For instance; a postpartum haemorrhage or manual removal of the placenta are both complications that have higher risks to be referred in the intrapartum period, however they are not leading to more medical problems in the current pregnancy. In 2015-17 a consultation with an obstetrician was necessary to arrange shared care during the delivery; a midwife (primary care) is responsible in the hospital and medical support of the obstetrician (secondary care) is accessible and nearby if necessary.  This might explain why multiparous women have consultations in the antepartum period which are not leading to more referrals during pregnancy, but only to more referrals during delivery. We have elaborated on this in the discussion part.  *“For instance; multiparous women with a history of postpartum haemorrhage or manual removal of the placenta needed a consultation during their current pregnancy. However, they were not at risk for medical complications in the antepartum period.”* | Page 18, lines 260-262 |
| Minor issues L 124 Maternal age is a continuous variable. Did you consider categorising this variable? There may well be a non-linear or (u-shaped) relation in your data, with higher complications and referrals for young (teens) and older women (> 35 or 40 yrs) | We have considered the option to categorise maternal age; however, most participants were between age 20 and 35 years, making the typical cut-off ages not suitable to have as categories. As described in the manuscript, we checked the assumption of linearity, which was not violated for any of the variables including maternal age. |  |
| L 144 Among the 617 women who started labour in midwifery led care, 287 (47%) were referred to obstetric-led care, 62% of nulliparous and 33% of multiparous women. In the introduction, you describe percentages of 21%, probably with another denominator (all women, instead of women who started labour in midwifery led care). This is somewhat confusing. | The percentages mentioned in the introduction are all the women registered in Perined. In our paper, we only focused on the women who started in midwife-led care. To compare our results with the Perined numbers, we recalculated the percentage of women who are referred after starting in the midwife-led care: in 2004, 41% (antepartum) and 27% (intrapartum) were referred to obstetrician-led care compared to 2016 when 40% (antepartum) and 41% (intrapartum) were referred. We added these numbers in the introduction.  *“In the Netherlands, the number of referrals in the intrapartum period has increased from 27% to 41% over the past 12 years.”* | Page 4, lines 82-84 |
| Table 1: consider to organise the columns differently and group them by parity. This makes it easier to compare women of the same parity without referral with women with referral. | We have changed the order of the columns; now first grouped by parity and then by referral. | Page 10-13 |
| Line 239 “We cannot explain the results as research has consistently shown that a history of sexual violence is associated with perinatal outcomes (41).” Reference 41 (van der Hulst ea, 2006 ) is perhaps not the best study to refer to, as it is not an example of a study that show this association. On the contrary, this study shows no important differences in referrals and birth-interventions for women with a history of sexual violence who were in primary midwifery care in the Netherlands. | We have reviewed reference 41 (van der Hulst ea, 2006), and it does not have these associations. We have carried out another literature review and concluded that these studies showed an association between a history of sexual abuse and more psychological problems and perinatal outcomes.  *“This is not consistent with the literature, which showed that a history of sexual abuse is associated with more psychological problems and adverse perinatal outcomes.”*  Added references: *Brunton R, Dryer R. Child Sexual Abuse and Pregnancy: A Systematic Review of the Literature. Child Abuse & Neglect. 2021;111:104802.*  *Dahlen HG, Munoz AM, Schmied V, Thornton C. The relationship between intimate partner violence reported at the first antenatal booking visit and obstetric and perinatal outcomes in an ethnically diverse group of Australian pregnant women: a population-based study over 10 years. BMJ Open. 2018;8(4):e019566.* | Page 19, lines 276-278 |
| *Reviewer 2* | | |
| An interesting paper that could feed the discussion on the quality of risk selection during pregnancy and birth in the Netherlands. Albeit with caution as the results are based on the data of one midwifery practice alone.  My main problem lies in the formulation of the research question. As I am not sure it is framed correctly. | We have changed the research question as follows: *“Therefore, our study investigated which maternal characteristics are associated with women’s referral from midwife-led to obstetrician-led care in the antepartum and intrapartum periods”.* | Page 5, lines 101-103 |
| The paper would benefit from textual and English language editing | One of the co-authors is fluent in English (Dr L.A. Horvat-Gitsels) and has been working as an academic for 10 years in England whilst publishing numerous peer-reviewed papers as the first author. She edited our manuscript. |  |
| L25 Rather bold opening statement, which might not be strictly true if this is meant world-wide. Perhaps add region / country. | We clarified this statement by adding “In the Netherlands” to the first sentences.  *“In the Netherlands, maternity care is divided into midwife-led (low-risk women) and obstetrician-led care (high-risk women). Referrals from midwives to obstetricians have increased.”* | Page 2, lines 25-27 |
| L27 Risk assessment is not only applied at the beginning of pregnancy but also during each prenatal check-up as well as during labour and post-partum period. | We have changed this sentence as follows:  *“**As risk assessment is a continuous process during pregnancy and childbirth most women are referred during these times.”* | Page 2, lines 27-28 |
| L27 Slightly vague: you mean maternal characteristics that influence referrals? | We have updated the research question and clarified this sentence in the text*.*  *“Risk assessment mainly focuses on medical characteristics, yet non-medical maternal ones remain unclear.”* | Page 2, lines 30-31 |
| L30 ‘indicate’ should be ‘influence’ or do you really mean a characteristic could be an indication for referral? | We have addressed the research question; the word “indicate” is replaced by the word “associate” | Page 3, lines 59-61 |
| L32 ‘a midwifery practice’ > better ‘one midwifery practice’ | We have adjusted this according to your suggestion. | Page 2, line 35 |
| L33 I am not sure what an outcome referral is, perhaps you mean primary outcome or outcome of referral(s)? | We mean that the primary outcome is a referral. We have clarified this by adding the word “primary outcome”. | Page 2, line 36 |
| L35 indicators for? | We have addressed the research question; the word “as possible indicators” have been removed. | Page 2, lines 31-33 |
| L39 obesity instead of obese | We have changed this according to your suggestion. | Page 2, lines 43 & 46 |
| L41 sexual abuse instead of sexual violence | We have adjusted this according to your suggestion. |  |
| L44 consultations with? The midwife? Obstetrician? (L48 / L50same) | This is a consultation with the obstetrician, we have added this to the sentence.  *“…one or multiple consultations with the obstetrician…”* | Page 3, lines 49 & 56 |
| L52-56 Conclusions: I think this conclusion cannot be made on the basis of this study and these results. The results show that certain characteristics seem to be associated with an increased referral rate but do not show if the referral was justified based on these characteristics. Although it could be argued that a referral of some sort might be needed or better more care and support, it does not necessarily mean an obstetric referral is the answer in order to improve outcomes. Therefore to conclude that these characteristics are an indicator for referral to secondary care is not correct. The study does not show whether a referral to secondary care on the basis if these characteristics influence outcomes of pregnancy.  What’s more I think caution with the conclusion, also or may be even more so in the abstract, would be prudent as the results are based on the data of one midwifery practice only and as we know from earlier research, referral rates vary greatly in the Netherlands. | This is an exploratory study in a highly diverse midwifery practice in the Netherlands. The results are informative of what other factors in addition to medical ones that are currently part of the risk assessment, are associated with a referral or increased need of care, and thus might need more attention by health care professionals, be it the GP, midwife, obstetrician, or psychologist.  We have made changes to the conclusion according to your suggestion:  *“This exploratory study showed that several non-medical maternal characteristics of low-risk pregnant women are associated with referral from midwife-led to obstetrician-led care, and how these differ by parity and partum period.”* | Page 3, lines 59-61 |
| L61: First sentence does not add anything: can be removed. Instead provide a bit more information on the different roles that midwives can have or the different systems that can be found. | We have removed the first sentence and added information on different care systems and the role of the midwife.  *“Midwife-led care is a model where “the midwife is the lead professional in the planning, organisation and delivery of care given to a woman from initial booking to the postnatal period” (2). Other models of care are obstetrician-provided care, family doctor-provided care or shared care.”* | Page 3, lines 70-73 |
| L73 Please add some information on referrals for pain relief. It will not be clear to all readers that the need for pharmacological pain relief is a reason for referral in the Netherlands and that the need / wish for this has been rising and therefore influences referral numbers. | We have added information as to why a referral is necessary for medical pain relief:  *“**The intrapartum indications include a request for medical pain relief such as epidural analgesia, presence of meconium-stained amniotic fluid, and failure to progress in the first or second stage of labour.* *Especially, the number of referrals for a request for medical pain relief has increased; in 2004 only 4% of the women received epidural analgesia during labour compared to 21% in 2017. Midwives are not qualified taking care of women who receive an epidural in the Netherlands, therefor they are referred to an obstetrician.”* | Page 4, lines 90-93 |
| L77 C-section > please write out in full at first use | We have corrected this by fully writing the word caesarean section. |  |
| L86-88 I am having problems with the word ‘indicator’. I am not certain what the authors mean by this: do they mean to investigate whether certain characteristics were used as an indicator for / of referral? Or did they investigate if certain characteristics gave women a higher chance of referral? Or if there were certain characteristics associated with referral from midwifery- to obstetric lead care? Or did the authors want to investigate whether certain characteristics are *predictors* for referral?  After reading the main analysis section, I think what is meant is characteristics which were associated with a lower / higher chance of referral. In that case: please adjust the research question.  Please adjust the title of the manuscript accordingly. | We have addressed these comments, please see the response to the previous reviewer's questions. (see page 2 and 7) |  |
| L94 I believe the word ‘received’ should perhaps be replaced by ‘continued primary care after the first / booking visit at the midwives practice’ , unless the sample also includes women who were referred from secondary to primary care at a later stage? | The sample also included women who were referred from secondary to primary care in the postnatal period; they did not start their care in the primary setting, because of their high-risk profile at the start of their pregnancy. Therefore, no changes were made to the manuscript. |  |
| L96/97 I am not sure what is meant by ‘noted in their files under their supervision’? | We meant that we have made a note in the medical file, during the consultation, so the woman was able to see this (under their supervision). This request was made by the Medical Ethics Committee. We have clarified it by adjusting the sentence:  *“Informed consent was obtained verbally and noted in their files in their presence”* | Page 5, lines 111-112 |
| L112 ‘summarised by’, should be ‘categorized in’ and reasons referred were (also?) based on the LOI? | We have changed the words into *“…were grouped by…”,* because it was not categorized.  And added the text*: “* *and based on regional protocols and the LOI”* | Page 6, lines 143-144 |
| L114 Could you add some information on how these data were obtained? Ie by a questionnaire? Did the midwife ask this a standard question during the booking visit? How was ethnicity, deprivation defined? And preconception period, ie what constitutes a preconception period of longer than a year, how or by whom defined? | *As described in the Method section, all information was obtained during the consultation with the midwife and noted in their files. We have added the definitions of ethnicity, deprivation and preconception period.*  *“Maternal non-medical characteristics were based on previous research on the effect of these characteristics on morbidity and mortality of mother and child. They were obtained from women’s medical files and information was collected by verbal questions of the midwife during the consultations with the woman. Dichotomous variables included: preconception period (≤1 year/>1 year) -- the period the woman was trying to conceive -- , employed (yes/no), ethnicity (Dutch/non-Dutch) --based on the birthplace of the mother or how they identified themself--, deprivation (no/yes) --living in a zip code area that is known to be deprived --, smoking (no/yes), psychological problems (no/yes), and sexual abuse (no/yes). Education level was categorised into low (baseline: primary education, pre-vocational secondary education or secondary vocational education), medium (senior general secondary education or pre-university education) and high (higher professional education or university education). BMI and number of consultations with an obstetrician were categorised due to non-normal distributions. Underweight was defined as a BMI <18.5, healthy weight was defined as a BMI 18.5-24.9 (baseline), overweight was defined as a BMI 25.0-29.9, and obesity was defined as a BMI ≥30.0. The number of consultations with an obstetrician --a standalone consult in obstetric care without women discontinuing midwife-led care-- was defined as none (baseline), one, and ≥2. Maternal age was a continuous variable, based on the date of birth.”* | Page 5 & 6, lines 119-135 |
| L120 ‘BMI was defined as underweight’ > should be phrased the other way around: Underweight was defined as a BMI < etc etc. | We have adjusted this according to your suggestion  *“Underweight was defined as a BMI <18.5, healthy weight was defined as a BMI 18.5-24.9 (baseline), overweight was defined as a BMI 25.0-29.9, and obesity was defined as a BMI ≥30.0.”* | Page 6, lines 131-132 |
| L123 maternal age based on DoB? | Yes, this is based on the date of birth, we added this to the sentence. | Page 6, line 135 |
| L142-146 please add that the data of 31 women were missing. | We have added this. | Page 7, line 165 |
| Table 1a & 1b: this shows the presence of certain characteristics in the group of women who entered primary care (1a) and women who started labour in primary care (1b) and you show the women with those characteristics who were not referred and who were referred: However you did not test for statistical differences between those groups. With other words: I would like to know if there was a difference in the chosen characteristics between the women who were referred and those who were not. | The intrapartum period group is a subgroup of the antepartum period. For differences between the groups, one can consult Table 1. No statistical tests were performed as this was not part of the research question (the objective was to estimate the association between maternal characteristics and referral by parity and partum period, which we did in the regression analyses). |  |
| L154-156 please add the stats for the comparison | We have added the statistics in the text:  *“**The proportion of missing data did not differ by referral and parity in the antepartum period; aOR of 0.86 (95%CI, 0.46-1.57) for nulliparous women and 1.34 (0.81-2.21) for multiparous women. However, it was higher among multiparous women who were referred in the intrapartum period (2.52; 1.25-5.13).”* | Page 13, lines 176-179 |
| Tables 2a & 2b: Please add a description of the outcomes that are written in bold text ( I think it means the outcomes that are significant: in that case, please check for mistakes) | We have added a description and corrected the mistake. | Page 14-16 |
| L204-206 the description of what the study investigated here is not in line with the research question. | We have addressed the research question. The description provided in the Discussion section is now in line with the research question. |  |
| L240 perinatal outcomes: which ones: positive or negative ones? | The perinatal outcomes are negative/adverse. We have added this to the sentence.  *“This is not consistent with the literature, which showed that a history of sexual abuse is associated with more psychological problems and adverse perinatal outcomes.”* | Page 19, lines 276-278 |
| L250-251 please move information on data collection to the methods section (also see earlier remark) | We have added this information in the method section.  *“They were obtained from women’s medical files and information was collected by verbal questions of the midwife during the consultations with the woman.”* | Page 5, lines 121-122 |
| L285 Our study shows that maternal characteristics > there are words missing > *the effect of certain* maternal characteristics etc | We have changed this part of the conclusion, because of, the comments of the reviewer(s). | Page 22, line 348-351 |
| Since the study was only performed in one midwifery practice and since we know that referral rates differ greatly between practices and regions, I would expect some reflection on this in the discussion. Possibly the characteristics of the midwives were of greater influence than those of the women… | We have addressed this issue in the discussion.  *“Nationally, the rate of referrals varies by practice and location. During the intrapartum period, referrals of nulliparous or multiparous women range between 55-68% and 20-32%, respectively (in our study 62% and 33%, respectively). The care providers' assessment of risk and uncertainty, as well as area guidelines, may impact the referral rate.”*  Added reference:  *Seijmonsbergen-Schermers AE, Zondag DC, Nieuwenhuijze M, van den Akker T, Verhoeven CJ, Geerts CC, Schellevis FG, de Jonge A. Regional variations in childbirth interventions and their correlations with adverse outcomes, birthplace and care provider: A nationwide explorative study. PLoS One. 2020 Mar 5;15(3):e0229488. doi: 10.1371/journal.pone.0229488. PMID: 32134957; PMCID: PMC7058301.* | Page 20, lines 299-304 |
| 288-291 I think this part of the conclusion should be worded more carefully as it is not clear that using these characteristics to determine / or screen for risk, and referring women to secondary care on the basis of this, improves outcomes.  Women with characteristics such as deprivation, unemployment, a history of sexual abuse might for example benefit from a different type of referral than that to secondary care. | We have adjusted the conclusion in the abstract and the conclusion in the last paragraph of the study.  *“Particularly characteristics such as deprivation, unemployment and a history of sexual abuse might benefit from* *other care systems or interventions such as case-load care or resilience enhancing interventions such as Centering Pregnancy, as well as supportive care of a welfare* worker or *psychologist. We advocate further research to increase awareness of the influence of all maternal characteristics**, as well as research about interventions that can improve modifiable maternal characteristics in the preconception and/or antepartum period.”* | Page 22, lines 351-358 |
| I don’t think figure 1 adds anything to the paper: I would leave it out. | Thank you for the recommendation, we have removed figure 1 from the manuscript. |  |
| 2a: since the item ‘other’ is in 4^th^ place: could you elaborate on this? The number of referrals for gestational diabetes seem quite large: how does this compare to (inter-) national numbers? | The group ’other’ were women with medical complications such as cholestasis, maternal heart defects, rheumatism, gastric bypass, or pelvic pain. We have added this in a footnote.  The number of referrals for gestational diabetes was during the period that all women were referred to obstetrician-led care if they had gestational diabetes, also when a related diet was in order. So, it is not representative of the current national policy. We have added this information to the discussion.  *“During our study period all women with gestational diabetes were referred to obstetrician-led care, also when only a diet was applied to stabilize their glucose values. Nowadays this policy is no longer in use in this region.”* | Page 20, lines 311-313 |
| 2b: I am surprised to see coagulation disorders and Uterus myomatosus as reasons for referral during labour as opposed to during pregnancy? | We agree that uterus myomatosus and coagulation disorder is an indication to refer during the pregnancy. During pregnancy, this complication is not observed and during the beginning of labour, it was noticed. We have combined these reasons in the group “others”. |  |
