# Supplementary material for: Maternal characteristics associated with referral to obstetrician-led care in low-risk pregnant women in the Netherlands: A retrospective cohort study
Source: PLoS One. 2023 Mar 15;18(3):e0282883. doi: 10.1371/journal.pone.0282883 (PMC10016726; doi:10.1371/journal.pone.0282883)
Supplement: Attachment — Submitted filename: Review-2 PONE_D-21-28982_juli 2022.docx [file pone.0282883.s006.docx]

This study is a descriptive study that shows which factors, other then official indications, are associated with referral to secondary care in a single midwifery practice.

Although the focus of the paper has not improved, in fact I find it more confusing, I do appreciate the change of tone in the paper: from talking about risk assessment in support of referrals, the discussion now pays attention to how midwifery care can be improved for women with certain characteristics.

My main issue with the paper remains that the focus is unclear and even confusing (also see later remark) and I wonder if and what it adds to the discussion on referral rates or quality of care.

The standard of English could still be improved. Please have it revised by a native speaker.

Other remarks:

L26 add time frame

L27 This is a not a logical sentence: the first part is not necessarily followed by latter part. I would just keep it simple: most women are referred during pregnancy or labour.

And then you explain about the system: Referrals are based on a continuous risk assessment which mainly focuses on etc.

L29. I disagree. It does not only or mainly focuses on characteristics. It focusses on health of mother and baby and development of pregnancy. You describe this better in the main paper.

L92: please be more specific to ensure correctness: Dutch primary care midwives are not qualified to care for women with epidural anesthesia etc

L83-85: I disagree. The non-medical factors are def. embedded in routine care. Ie midwives routinely ask / talk about lifestyle factors such as smoking, alcohol use / drugs etc. BMI is noted on intake. And a considerable amount of midwives are nowadays aware of the importance of asking about Negative sexual experiences. Etc etc. However these factors are not part of the official nationwide referral system > that is a different thing.

L85 despite their association with negative perinatal outcomes

L85-90: I find this very confusing. What are you trying to say, what is your focus? My impression is that you want to look at too many issues at the same time or that your focus is not clear.

Why do we need to know which non-medical factors are increasing a referral (you mean the chance of a referral?) in order to be able to intervene? Is it not possible to intervene without referral?

Ie stop-smoking support can very well be carried out in primary care, the same with weight loss and diet. And I doubt that secondary or tertiary care can do anything about social conditions such as housing or financial problems.

Or do you want to gain more knowledge about factors that (?subconsciously) influence a midwives decision to refer: if so I wonder if the used methodology is the correct one to answer this question.

Or do you want to know which non-medical factors influence referral as this may influence perinatal outcomes?

L103—120 : please indicate primary and secondary outcomes

L105 And I think you mean: Secondary outcomes such as maternal non-medical characteristics were chosen based on the literature etc.

And please clearly describe what your baseline characteristics are. (this is not clear here).

Data were obtained from women’s medical records etc etc.

L106 and information was collected > which information ?

L109-111 based on the birthplace of the mother

125 or how they identified themself--, deprivation (no/yes) --living in a zip code area that is known to be deprived.

PLease rewrite this sentence as it is very confusing . Moreover: the birthplace (or do you mean country of birth?)of the mother determined deprivation status? How so? Please explain and substantiate this.

L144-145. 617 women started labour in primary care but were migrated at end of antepartum period: how is this possible? Or what do you mean with “migrated”? Please explain or clarify.

L207 Are of importance for what?

L209 > what do you mean with preconception period? The length of preconception period? Please clarify.

L224-224 What about the influence of different(views of) obstetricians (inter- observervariability) on care? (this also counts for midwives of course)

L269-270 Yes this true but what does it have to do with referral rates? This is a complex discussion which is not adequately touched.

L273 etc Please finish the sentence; Centering pregnancy facilitates social and group support > so? What does this mean? Do you think more women should have the opportunity to participate in this model of care?

L277-278 Why do you want to do this? You want to improve quality of care? Improve the quality of referrals?
