# Supplementary material for: Maternal characteristics associated with referral to obstetrician-led care in low-risk pregnant women in the Netherlands: A retrospective cohort study
Source: PLoS One. 2023 Mar 15;18(3):e0282883. doi: 10.1371/journal.pone.0282883 (PMC10016726; doi:10.1371/journal.pone.0282883)
Supplement: Attachment — Submitted filename: Response to Reviewers.docx [file pone.0282883.s007.docx]

August 25, 2022

Manuscript ID: PONE-D-21-28982R1

Dear Editor and reviewers,

Thank you for the expert review of our revised manuscript titled ‘Maternal characteristics associated with referral to obstetrician-led care in low-risk pregnant women in the Netherlands: a retrospective cohort study’.

We want to thank you and the reviewers for the second round of comments. We have used the reviewers' comments to improve our paper further, and we are pleased to send you our revised manuscript. The reviewers' comments and our responses are presented in the table below. The actual changes in the paper are presented in track changes. Furthermore, as suggested by Reviewer 2, a native speaker revised the English language.

Sincerely, on behalf of all authors,

Susan Niessink-Beckers

| **Reviewers’ comment** | **Authors’ response** | **Changes in manuscript with tracked changes** |
| --- | --- | --- |
| *Reviewer 1* | | |
| Line 80: insert: "Primary care midwives" instead of) 'Midwives' are not qualified taking care of women who receive an epidural in the Netherlands, therefor they are referred to obstetrician-led care (instead of "the obstetrician'). | We have adjusted this according to your suggestion:  *Dutch primary care midwives are not qualified to care for women who receive an epidural in the Netherlands. Therefore, they are referred to obstetrician-led care.* | P4,L83-85 |
| Line 87 To be able to intervene on those factors, we need to know which kind of non-medical factors are increasing (Insert: "the chance of") referral to obstetrician-led care | We have adjusted this according to your suggestion:  *Before we can intervene on those factors, we need to know which non-medical factors increase the likelihood of referral to obstetrician-led care.* | P4, L93-95 |
| Line 172; table 2a/2b Please replace ‘Indicators for referral’ with "association with referral " | We have adjusted this according to your suggestion:  *See Table 2a/2b.* | P11, L184 |
| Line 196 Remove the ; in this line after "or they had ; " | We have removed this according to your suggestion.  *Women were less likely to be referred when their preconception period was more than one year (0.42; 0.31-0.57) or they had a history of sexual abuse (0.46; 0.33-0.63).* | P14, L 206-208 |
| Lin2 271 “Lack of social support is associated with adverse perinatal outcomes and should be considered as an opportunity to intervene on psychosocial and lifestyle maternal characteristics [48, 49].” This is a bit confusing. I suggest splitting this sentence and changing it a little into: Lack of social support is associated with adverse perinatal outcomes. For these women, pregnancy should be considered …etc. | We have adjusted this sentence as follows:  *Non-medical issues could be addressed at an earlier stage, preferably during preconception or at the beginning of pregnancy. For instance, addressing the lack of social support is important during pregnancy, as this is associated with adverse perinatal outcomes [48, 49].* | P17, L289-292 |
| Line 287 “care systems” seems not the appropriate concept, I suggest “care models” . | We have adjusted this according to your suggestion:  *In particular, certain characteristics such as deprivation, unemployment and a history of sexual abuse might benefit from other care models or interventions. These include case-load care or resilience-enhancing interventions such as CenteringPregnancy, as well as the supportive care of a welfare worker or psychologist.* | P18, L314-317 |
| Line 289: Do you really mean “all characteristics” or do you want to focus on non-medical modifiable characteristics (since medical characteristics already have the attention of maternity care professionals) ? | Thank you for the suggestion. We clarified it to the following:  *We advocate further research to increase awareness of the influence non-medical characteristics have on referral, as well as research about interventions that could improve modifiable maternal characteristics in the preconception and/or antepartum period.* | P18, L317-320 |
| *Reviewer 2* | | |
| My main issue with the paper remains that the focus is unclear and even confusing (also see later  remark) and I wonder if and what it adds to the discussion on referral rates or quality of care. | Thank you for bringing this to our attention. We have improved our manuscript, taking your concerns into account. |  |
| The standard of English could still be improved. Please have it revised by a native speaker. | The manuscript was reviewed and improved by Dr Vicky S Adams, a native speaker. |  |
| L26 add time frame | We have added the time frame in the text.    *Referrals from midwife-led to obstetrician-led care have increased over the past decade.* | P2, L26-27 |
| L27 This is a not a logical sentence: the first part is not necessarily followed by latter part. I would just  keep it simple: most women are referred during pregnancy or labour.  And then you explain about the system: Referrals are based on a continuous risk assessment which  mainly focuses on etc. | We have adjusted this according to your suggestion.  *The majority of women are referred during their pregnancy or labour. Referrals are based on a continuous risk assessment of the* *health and characteristics of mother and child, yet referral for non-medical factors and characteristics remain unclear.* | P2, L27-31 |
| L29. I disagree. It does not only or mainly focuses on characteristics. It focusses on health of mother  and baby and development of pregnancy. You describe this better in the main paper. | Referrals are based on the characteristics and the medical health issues. We have added this to the text.  *Referrals are based on a continuous risk assessment of the health and characteristics of mother and child, yet referral for non-medical factors and characteristics remain unclear.* | P2, L 28-31 |
| L92: please be more specific to ensure correctness: Dutch primary care midwives are not qualified to  care for women with epidural anesthesia etc | We have adjusted this according to your suggestion:  *Dutch primary care midwives are not qualified to care for women who receive an epidural in the Netherlands. Therefore, they are referred to obstetrician-led care.* | P4, L 83-85 |
| L83-85: I disagree. The non-medical factors are def. embedded in routine care. Ie midwives routinely  ask / talk about lifestyle factors such as smoking, alcohol use / drugs etc. BMI is noted on intake. And a considerable amount of midwives are nowadays aware of the importance of asking about Negative  sexual experiences. Etc etc. However these factors are not part of the official nationwide referral  system &gt; that is a different thing.  L85 despite their association with negative perinatal outcomes | Indeed, during the first consultation midwives ask about the lifestyle factors. We removed the following sentence: “however, maternal characteristics such as psycho-social and lifestyle factors, also known as non-medical factors, are not embedded in routine risk assessment, despite their associations with perinatal outcomes” and now reads as follows:    *Numerous studies have determined the influence of risk factors such as body mass index (BMI) or maternal age on perinatal outcomes [13-17]. However, few studies have investigated the association between perinatal outcomes and maternal characteristics on non-medical factors, such as psychosocial and lifestyle factors [18-25].* | P4, L86-89 |
| L85-90: I find this very confusing. What are you trying to say, what is your focus? My impression is  that you want to look at too many issues at the same time or that your focus is not clear.  Why do we need to know which non-medical factors are increasing a referral (you mean the chance  of a referral?) in order to be able to intervene? Is it not possible to intervene without referral?  Ie stop-smoking support can very well be carried out in primary care, the same with weight loss and  diet. And I doubt that secondary or tertiary care can do anything about social conditions such as  housing or financial problems.  Or do you want to gain more knowledge about factors that (?subconsciously) influence a midwives  decision to refer: if so I wonder if the used methodology is the correct one to answer this question.  Or do you want to know which non-medical factors influence referral as this may influence perinatal  outcomes? | The purpose (main goal) of our study was to investigate which non-medical factors are associated with referrals. Some lifestyle factors such as a higher BMI or age are associated with adverse perinatal outcomes and therefore women with these characteristics have a higher chance of being referred. For other non-medical factors such as a history of sexual violence or preconception period, the evidence is limited. Therefore, our study investigated the relationship between non-medical risk factors and the association of non-medical factors with referral. This knowledge could be useful in developing possible interventions for intervening during the preconception or prenatal period by applying a different care model or introducing a caregiver who is specialized in the subject, for instance a psychologist. This information could help in creating the individualized preventive care.  *Numerous studies have determined the influence of risk factors such as body mass index (BMI) or maternal age on perinatal outcomes [13-17]. However, few studies have investigated the association between perinatal outcomes and maternal characteristics on non-medical factors, such as psychosocial and lifestyle factors [18-25]. Awareness of all maternal characteristics, including non-medical factors affecting the chance of a referral, will help healthcare professionals provide individualised preventive care [26, 27].* *Before we can intervene on those factors, we need to know which non-medical factors increase the likelihood of referral to obstetrician-led care. Therefore, our study investigated which non-medical maternal characteristics are associated with women’s referral from midwife-led to obstetrician-led care in the antepartum and intrapartum periods, as these may influence perinatal outcomes.* | P4, L86-97 |
| L103—120 : please indicate primary and secondary outcomes  L105 And I think you mean: Secondary outcomes such as maternal non-medical characteristics were  chosen based on the literature etc. | Our two primary outcomes were a referral from midwife-led to obstetrician-led care in (1) the antepartum (yes/no) and, (2) the intrapartum period (yes/no). Our independent variables of interest were maternal non-medical characteristics. Our study investigated to what extent these characteristics were associated with the outcomes of referral during pregnancy and labour. We clarified this in the manuscript:  *The two primary outcomes were defined as a referral from midwife-led to obstetrician-led care in the antepartum (yes/no) and, given no previous referral, in the intrapartum period (yes/no). Maternal non-medical characteristics - the independent variables of interest - were based on literature on the effect of these characteristics on morbidity and mortality of mother and child [13, 15-18, 21, 23-25, 28].* | P5, L111-114 |
| And please clearly describe what your baseline characteristics are. (this is not clear here). | The baseline characteristics (including the medical and non-medical factors which are the independent variables of interest) are described in the Measures in the Materials and Methods section and presented in Table 1. We added the following sentences:  *Maternal non-medical characteristics - the independent variables of interest - were based on literature on the effect of these characteristics on morbidity and mortality of mother and child [13, 15-18, 21, 23-25, 28]. These characteristics were obtained from the women’s medical records where they were noted by the midwife at the beginning of the antepartum period. Dichotomous variables included: preconception period (≤1 year/>1 year) --the period the woman was trying to conceive--, employment (yes/no), ethnicity (Dutch/non-Dutch) --based on the mothers country of birth --, deprivation (no/yes) -- based on a zip code classified as a deprived area[29]--, smoking (no/yes), psychological problems (no/yes), and sexual abuse (no/yes). Education level was categorised into low (primary education, pre-vocational secondary education or secondary vocational education), medium (senior general secondary education or pre-university education) and high (higher professional education or university education). BMI and number of consultations with an obstetrician were categorised due to non-normal distributions. Underweight was defined as having a BMI of <18.5, healthy weight was defined as a BMI of 18.5-24.9, overweight was defined as a BMI of 25.0-29.9, and obesity was defined as a BMI of ≥ 30.0 [30]. The number of consultations with an obstetrician --a standalone consult in obstetric care without women discontinuing midwife-led care-- was defined as none, one, and ≥2. Maternal age was a continuous variable based on the date of birth.* | P5-6, L112-130 |
| Data were obtained from women’s medical records etc etc.  L106 and information was collected &gt; which information ? | “Information” refers to the non-medical characteristics. We have adjusted this as follows:  *These characteristics were obtained from the women’s medical records where they were noted by the midwife at the beginning of the antepartum period.* | P5, L 115-116 |
| L109-111 based on the birthplace of the mother  125 or how they identified themself--, deprivation (no/yes) --living in a zip code area that is known to  be deprived.  PLease rewrite this sentence as it is very confusing . Moreover: the birthplace (or do you mean  country of birth?)of the mother determined deprivation status? How so? Please explain and substantiate this. | We have rewritten this text as follows:  *Dichotomous variables included: preconception period (≤1 year/>1 year) --the period the woman was trying to conceive--, employment (yes/no), ethnicity (Dutch/non-Dutch) --based on the mothers country of birth --, deprivation (no/yes) -- based on a zip code classified as a deprived area[29]--, smoking (no/yes), psychological problems (no/yes), and sexual abuse (no/yes).* | P5, L117-122 |
| L144-145. 617 women started labour in primary care but were migrated at end of antepartum  period: how is this possible? Or what do you mean with “migrated”? Please explain or clarify. | We mean that they moved to another city or country, so they did not finish the care at the same practice. We have clarified this in the text as follows:  *Among the 617 women who started labour in midwife-led care, 31 (5%) were moved to another practice or clinic in the country by the end of the antepartum period […].* | P7, L154-157 |
| L207 Are of importance for what? | Those characteristics are associated with referral in the antepartum and intrapartum periods, so they are of importance in influencing the referral rate. We have adjusted this as following:  *In addition to the more commonly researched maternal characteristics such as BMI and age, we showed that non-medical characteristics such as employment, education level, history of sexual abuse and consultations in obstetrician-led care are associated with referral, and differ by parity and partum period.* | P14, L220-224 |
| L209 &gt; what do you mean with preconception period? The length of preconception period? Please  clarify. | We mean the length of the preconception period. We have described this in the Methods section. | P5, L117-122 |
| L224-224 What about the influence of different(views of) obstetricians (inter- observervariability) on  care? (this also counts for midwives of course) | The view of an obstetrician and also a midwife can influence the care given, however this applies in general and not only for the number of consultations in the obstetrician-led care. We have mentioned this as one of the limitations in the discussion:  *Our low-risk study population included only one midwifery practice. Nationally, the rate of referrals varies by practice and location. A nationwide retrospective cohort study reported that intrapartum referrals of nulliparous or multiparous women range between 55-68% and 20-32%, respectively (in our study 62% and 33%, respectively). The care providers' assessment of risk and uncertainty, as well as regional guidelines, may impact the referral rate [43]. Therefore, we cannot generalise our results to all low-risk women in the Netherlands.* | P16, L272-277 |
| L269-270 Yes this true but what does it have to do with referral rates? This is a complex discussion  which is not adequately touched. | Referrals are increasing and thereby medical interventions/issues. To prevent medical issues and improve quality of care, we need to be aware of the association between the maternal characteristic and the referral rate. Then we are able to investigate preventive methods to reduce these referrals by improving the medical health of the woman and child. We have adjusted this as follows:  *Our study showed that multiple maternal characteristics are associated with a referral to obstetrician-led care. In particular, there are a number of non-medical characteristics associated with a referral that can affect the course of pregnancy and birth, but these are currently not considered in maternity care [26, 27]. Non-medical issues could be addressed at an earlier stage, preferably during preconception or at the beginning of pregnancy. For instance, addressing the lack of social support is important during pregnancy, as this is associated with adverse perinatal outcomes [48, 49]. Therefore, we would advocate more facilities for midwives, as they often provide close care to women and know their social environment. This would allow midwives to treat women with non-medical factors more intensely.*  *Care models such as midwife-led care, which emphasise continuity of care, are in themselves important for the well-being of the mother and child [1]; and other midwife-led care models, such as CenteringPregnancy (group sessions), facilitate social support and enable women to improve their self-confidence [50]. Continuous care might be beneficial for non-medical maternal characteristics, particularly since case-load midwifery care is associated with a lower referral rate [51]. Support from other caregivers is also accessible in the midwife-led care model, whereby the midwife can support women with the help of a psychologist, dietitian, or welfare worker. As preventive care focused on non-medical issues may benefit medical care, we advocate further research into the relationship between non-medical maternal characteristics and referral, as well as how midwives can improve midwife-led care for women with psychosocial or lifestyle issues (non-medical characteristics).* | P17-18, L285-308 |
| L273 etc Please finish the sentence; Centering pregnancy facilitates social and group support &gt; so?  What does this mean? Do you think more women should have the opportunity to participate in this  model of care? | CenteringPregnancy is a care model with an emphasis on social support and self-confidence and could be used as an intervention to address non-medical issues. Therefore women should have the opportunity to participate in this care model. We have adjusted this as follows:  *Care models such as midwife-led care, which emphasise continuity of care, are in themselves important for the well-being of the mother and child [1]; and other midwife-led care models, such as CenteringPregnancy (group sessions), facilitate social support and enable women to improve their self-confidence [50]. Continuous care might be beneficial for non-medical maternal characteristics, particularly since case-load midwifery care is associated with a lower referral rate [51]. Support from other caregivers is also accessible in the midwife-led care model, whereby the midwife can support women with the help of a psychologist, dietitian, or welfare worker. As preventive care focused on non-medical issues may benefit medical care, we advocate further research into the relationship between non-medical maternal characteristics and referral, as well as how midwives can improve midwife-led care for women with psychosocial or lifestyle issues (non-medical characteristics).* | P17-18, L295-308 |
| L277-278 Why do you want to do this? You want to improve quality of care? Improve the quality of  referrals? | We want to improve the quality of care. However our study showed associations between non-medical characteristics and referral. Women with non-medical issues might benefit from different care models. For instance, CenteringPregnancy or case-load care could improve the lack of social support. Therefore, we advocate further research into the relation between non-medical maternal characteristics and referral and how this relationship can improve midwifery-led care for women with non-medical issues. We have addressed this in the practical implications and future research section:  *Non-medical issues could be addressed at an earlier stage, preferably during preconception or at the beginning of pregnancy. For instance, addressing the lack of social support is important during pregnancy, as this is associated with adverse perinatal outcomes [48, 49]. Therefore, we would advocate more facilities for midwives, as they often provide close care to women and know their social environment. This would allow midwives to treat women with non-medical factors more intensely.*  *Care models such as midwife-led care, which emphasise continuity of care, are in themselves important for the well-being of the mother and child [1]; and other midwife-led care models, such as CenteringPregnancy (group sessions), facilitate social support and enable women to improve their self-confidence [50]. Continuous care might be beneficial for non-medical maternal characteristics, particularly since case-load midwifery care is associated with a lower referral rate [51]. Support from other caregivers is also accessible in the midwife-led care model, whereby the midwife can support women with the help of a psychologist, dietitian, or welfare worker. As preventive care focused on non-medical issues may benefit medical care, we advocate further research into the relationship between non-medical maternal characteristics and referral, as well as how midwives can improve midwife-led care for women with psychosocial or lifestyle issues (non-medical characteristics).* | P17-18, L289-308 |
