# Supplementary material for: Maternal characteristics associated with referral to obstetrician-led care in low-risk pregnant women in the Netherlands: A retrospective cohort study
Source: PLoS One. 2023 Mar 15;18(3):e0282883. doi: 10.1371/journal.pone.0282883 (PMC10016726; doi:10.1371/journal.pone.0282883)
Supplement: Attachment — Submitted filename: Review 3_PONE-D-21-28982R2_Maternal characteristics associated with obstetric referral.docx [file pone.0282883.s008.docx]

**Maternal characteristics associated with referral to obstetrician-led care in low-risk pregnant women in the Netherlands: a retrospective cohort study**

Thank you for sending me this much improved paper on the study of maternal characteristics in primary midwifery care. I would really like to compliment the authors. The text reads much better and I am especially pleased with the improvements in the discussion and conclusion.

I still have a few minor remarks that I trust won’t take much effort to adjust:

Page 4, last Alinea of introduction: Your focus and your reasons for wanting to carry out this study are now much better explained. However I still have some trouble with the first 2 sentences of this Alinea, they seem to be contradictory. Are lifestyle factors (mentioned in the 2^nd^ sentence) not factors such as BMI and maternal age? And there have been quite few studies so far that have shown an association between stress (psychosocial) and perinatal outcomes.

Moreover l87: …and maternal characteristics on non-medical factors > I think it should be: … and non-medical maternal characteristics?

P5 L101: I think there are words missing after “and”: Those who had a miscarriage were excluded and were referred to obstetrician-led care in the first trimester etc. This should read: and those who were referred to obstetrician-led care

P5 115/116: please change psychological problems into past (or previous) or current psychological problems and sexual abuse into a history of sexual abuse

P10, L172 Please finish the sentence … whilst those who were overweight or obese were more likely to be referred.

P12 L185 … and lived in deprivation. You do not know if they actually lived in deprivation, this is an assumption based on their zip/postal code. I suggest this should be changed to …and lived in a deprived area.

P15 L 268-270: During our study period, all women with gestational diabetes were referred to obstetrician-led care, when only a diet was applied to stabilise their glucose values. This sentence seems to have words missing after the 2^nd^ comma? > I think you need to insert the following words: …apart from when only a diet etc etc.

P16 L 274-275: Please be aware of the limitations of your study. Therefor insert “in this particular midwifery practice.” At the end of the sentence.

P16 L286: I think you mean continuity of care instead of Continuous care?

P16 L286-287: is this also the case for women with the described characteristics in your study? Do we know this?
