# Supplementary material for: Maternal characteristics associated with referral to obstetrician-led care in low-risk pregnant women in the Netherlands: A retrospective cohort study
Source: PLoS One. 2023 Mar 15;18(3):e0282883. doi: 10.1371/journal.pone.0282883 (PMC10016726; doi:10.1371/journal.pone.0282883)
Supplement: Attachment — Submitted filename: Response to Reviewers.docx [file pone.0282883.s009.docx]

February 6, 2023

Manuscript ID: PONE-D-21-28982R3

Dear Editor and reviewers,

Thank you for the expert review of our revised manuscript entitled ‘Maternal characteristics associated with referral to obstetrician-led care in low-risk pregnant women in the Netherlands: a retrospective cohort study’.

We want to thank you and the reviewers for the suggestions. We have used the reviewers' comments to improve our paper further, and we are pleased to send you our revised manuscript. The reviewers' comments and our responses are presented in the table below. The actual changes in the paper are presented in track changes.

Sincerely, on behalf of all authors,

Susan Niessink-Beckers

| **Reviewers’ comment** | **Authors’ response** | **Changes in manuscript with tracked changes** |
| --- | --- | --- |
| *Reviewer 1* | | |
| would you please check the subtitles in table 1a and 1b, where the % in the columns is explained? | We checked the subtitles in tables 1a and 1b and corrected these where needed. | P7-8, L 156 |
| *Reviewer 2* |  |  |
| Page 4, last Alinea of introduction: Your focus and your reasons for wanting to carry out this study are now much better explained. However I still have some trouble with the first 2 sentences of this Alinea, they seem to be contradictory. Are lifestyle factors (mentioned in the 2^nd^ sentence) not factors such as BMI and maternal age? And there have been quite few studies so far that have shown an association between stress (psychosocial) and perinatal outcomes. | The term lifestyle can be contradicting, therefore we have adjusted the sentences.  There are studies that have shown associations between stress (psychosocial) and perinatal outcomes. Our study does not investigate the association between stress and perinatal outcome, but investigates other psychosocial factors such as psychological problems or sexual abuse and its relation to a referral during the antepartum or intrapartum period. We have clarified this as follows:  *However, few studies have investigated the association between perinatal outcomes and a wide range of maternal characteristics that are readily available in clinical records, such as a history or presence of psychological problems or sexual abuse [18-25].* *Moreover, the associations between specifically non-medical maternal factors and referral towards obstetric care are still unknown.* | P4, L 86-90 |
| Moreover l87: …and maternal characteristics on non-medical factors > I think it should be: … and non-medical maternal characteristics? | We corrected the sentence based on your comment above:  *However, few studies have investigated the association between perinatal outcomes and a wide range of maternal characteristics that are readily available in clinical records, such as a history or presence of psychological problems or sexual abuse [18-25]. Moreover, the associations between specifically non-medical maternal factors and referral towards obstetric care are still unknown.* | P4, L 86-90 |
| P5 L101: I think there are words missing after “and”: Those who had a miscarriage were excluded and were referred to obstetrician-led care in the first trimester etc. This should read: and those who were referred to obstetrician-led care | We have adjusted this as you suggested:  *Those who had a miscarriage were excluded and those who were referred to obstetrician-led care in the first trimester or received only postnatal care.* | P5, L 103-104 |
| P5 115/116: please change psychological problems into past (or previous) or current psychological problems and sexual abuse into a history of sexual abuse | We have adjusted this as you suggested:  *Dichotomous variables included: preconception period (≤1 year/>1 year) --the period the woman was trying to conceive--, employment (yes/no), ethnicity (Dutch/non-Dutch) --based on the mothers country of birth --, deprivation (no/yes) -- based on a zip code classified as a deprived area[29]--, smoking (no/yes), psychological problems (past or current) (no/yes), and a history of sexual abuse (no/yes).* | P5, L 115-122 |
| P10, L172 Please finish the sentence … whilst those who were overweight or obese were more likely to be referred. | We have adjusted this as you suggested:  *Compared to women of healthy weight, those who were underweight were less likely to be referred (0.45; 0.31-0.64), whilst those who were overweight or obese were more likely to be referred (2.29; 1.91-2.74 or 2.65; 2.06-3.42, respectively).* | P10, L 174-178 |
| P12 L185 … and lived in deprivation. You do not know if they actually lived in deprivation, this is an assumption based on their zip/postal code. I suggest this should be changed to …and lived in a deprived area. | We have adjusted this as you suggested:  *Women were also more likely to be referred if their preconception period was longer than one year (1.71; 1.27-2.28), they had a history of psychological problems (1.24; 1.06-1.44), they worked during pregnancy (1.38; 1.19-1.61), and lived in a deprived area (1.23; 1.03-1.46).* | P12, L 185-189 |
| P15 L 268-270: During our study period, all women with gestational diabetes were referred to obstetrician-led care, when only a diet was applied to stabilise their glucose values. This sentence seems to have words missing after the 2^nd^ comma? > I think you need to insert the following words: …apart from when only a diet etc etc. | Thank you for your suggestion. It is in fact the other way around: women who only needed a diet to stabilise their glucose values were referred to obstetrician-led care during our study period. However, current practice in this region is to not refer them. We have clarified that in the manuscript:  *During our study period, all women with gestational diabetes were referred to obstetrician-led care.* *The current policy in this region is that women who only need a diet to stabilise their glucose values are not referred to obstetrician-led care.* | P16, L 272- 273 |
| P16 L 274-275: Please be aware of the limitations of your study. Therefor insert “in this particular midwifery practice.” At the end of the sentence. | We have adjusted this as you suggested:  *Our study showed that multiple maternal characteristics are associated with a referral to obstetrician-led care in this particular midwifery practice.* | P16, L 279-282 |
| P16 L286: I think you mean continuity of care instead of Continuous care? | We have adjusted this as you suggested:  *Continuity of care might be beneficial for non-medical maternal characteristics, particularly since case-load midwifery care is associated with a lower referral rate [51].* | P16, L 291 - 292 |
| P16 L286-287: is this also the case for women with the described characteristics in your study? Do we know this? | Yes, women with the described characteristics in our study had access to other caregivers. We have clarified that in the manuscript:  *The women in this study had access to other caregivers.* | P16, L291 |
